# Topical Umbilical Cord Serum for Corneal Epithelial Defects after Diabetic Vitrectomy

**DOI:** 10.18502/jovr.v16i1.8264

**Published:** 2021-01-20

**Authors:** Arjun Srirampur

**Affiliations:** ^1^Anand Eye Institute, Habsiguda, Hyderabad, India

Dear Editor,

I have read with great interest the article by Moradian et al on “Topical Umbilical Cord Serum for Corneal Epithelial Defects after Diabetic Vitrectomy.”^[1]^ They have performed the study to investigate whether topical umbilical cord serum (TUCS) has any beneficial role in healing corneal epithelial defects (CED) after diabetic vitrectomy. However, I have a few concerns about the study.

Firstly, the postoperative intraocular pressures (IOP) were not mentioned in both groups. Elevated IOP (which is common after a vitreoretinal surgery) is known to cause corneal edema and increase the risk of corneal complications such as epithelial defect and non- healing epithelial defect.^[2]^ It would be more informative if the authors had provided IOP measurements in both groups, as it could influence the healing pattern of the epithelial defects.

Secondly, it is mentioned that all 80 eyes underwent deep vitrectomy but it is not mentioned what type of intraocular tamponade agent such as air, Sulphur hexafluoride (SF6), perfluoropropane (C3F8), or silicone oil was used during the surgery. Intraocular tamponade of long-acting expansile gases may induce corneal endothelial cell toxicity. The loss of corneal endothelial cells has also been reported to be significantly greater in eyes with C3F8 than in those with SF6.^[3]^ This endothelial damage especially in a diabetic eye can lead to corneal edema and loose adhesions between the Bowman's layer and stroma which delays the epithelial healing mechanism.

Thirdly, even though the authors have admitted for the absence of dry eye testing in these eyes, they should have performed simple corneal sensations. It is well-known that diabetic eyes have corneal hypoesthesia due to peripheral neuropathy. The development of diabetic keratopathy has been suggested to be related to loss of nerve-derived trophic factors following a decrease in corneal sensation. This reduced corneal sensations can lead to neurotrophic keratopathy which can lead to disturbance in the healing of the epithelial defects. Diabetic keratopathy may be associated with neuropathic keratitis in patients with a persistent corneal epithelial defect, and dry eyes may occur secondary to a reflex decrease of tear secretion due to corneal hypoesthesia and/or secondary to reduced tear and mucin secretion due to efferent nerve dysfunction.^[4]^

Also, the risk of allergies and possibilities of transmitting parenterally transmitted organisms must also be kept in mind when using TUCS apart from the legal and ethical issues. A routine testing of the mothers and a rapid test on the sera for viral contaminants is required. When the event to the time between the testing of the mother for HIV and the preparation of cord serum from the placental blood is more than six months, HIV testing should be undertaken again at the time of serum delivery to account for the window period of the infection.^[5]^ In consideration of the window period of HIV infection, additional HIV testing with a shortened window period, such as p24 antigen detection method, should be performed before the use of the TUCS.
